# Mapping Treatment Advances in the Neurobiology of Binge Eating Disorder: A Concept Paper

**DOI:** 10.3390/nu16071081

**Published:** 2024-04-07

**Authors:** Brooke Donnelly, Phillipa Hay

**Affiliations:** 1 School of Psychology, Translational Health Research Institute (THRI), Western Sydney University, Sydney, NSW 2751, Australia; b.donnelly@westernsydney.edu.au; 2 School of Medicine, Translational Health Research Institute (THRI), Western Sydney University, Sydney, NSW 2560, Australia; 3 Mental Health Services, South Western Sydney Local Health District, Campbelltown, NSW 2560, Australia

**Keywords:** eating disorder, binge eating disorder, neurobiology, genome, gut microbiota, treatment

## Abstract

Binge eating disorder (BED) is a complex and heritable mental health disorder, with genetic, neurobiological, neuroendocrinological, environmental and developmental factors all demonstrated to contribute to the aetiology of this illness. Although psychotherapy is the gold standard for treating BED, a significant subgroup of those treated do not recover. Neurobiological research highlights aberrances in neural regions associated with reward processing, emotion processing, self-regulation and executive function processes, which are clear therapeutic targets for future treatment frameworks. Evidence is emerging of the microbiota-gut-brain axis, which may mediate energy balance, high-lighting a possible underlying pathogenesis factor of BED, and provides a potential therapeutic strategy.

## 1. Introduction

Eating disorders (EDs) are complex and heritable mental health disorders, often characterised by chronicity and relapse [1,2]. Internationally, binge eating disorder (BED) is the second most common type of ED following Other Specified Feeding and Eating Disorder (OSFED), with a global lifetime prevalence of around 2.8% in women and 1.0% in men [3,4]. Recurrent and compulsive binge eating is a core diagnostic symptom of EDs seen transdiagnostically in bulimia nervosa (BN), binge eating disorder (BED), anorexia nervosa binge–purge type (AN-BP) and is a common feature in other specified feeding and eating disorder (OSFED) [5].

The principal feature of BED is recurrent objective binge episodes (OBEs) which are defined as consuming an amount of food that is definitely larger than what would usually be eaten for the social or cultural context, coupled with a sense of a loss of control [5]. For a diagnosis of BED, OBEs must occur at least weekly for a minimum period of three months [5]. Unlike bulimia nervosa (BN), individuals with BED do not engage in compensatory behaviours following a binge episode, such as self-induced vomiting or excessive exercise. BED typically arises in later adolescence and presents significant social, financial and health consequences, such as impaired quality of life and impaired physical health [6]. Furthermore, whilst there remains some uncertainty and inconsistences in the epidemiological literature, a sequential population-based survey in Australia confirmed that binge eating has been increasing in the general community [7].

BED is also found in minority groups, such as Aboriginal and Torres Strait Islander populations in Australia [8] and Indigenous and African American populations in the United States [9]. These groups are vastly underrepresented in the research of eating disorders, including BED, and there are major racial disparities in treatment access for culturally and linguistically diverse groups. However, while limited evidence exists in the area of BED cross-culturally, the available evidence strongly suggests that eating disorders, including BED, are more common in these cultural groups partly due to poorer psychosocial wellbeing [8,9]. Data from population-based studies in high- and middle-income countries highlight that only approximately one-third of people have sought or received appropriate treatment [10]. Lower rates of access to evidence-based treatment and care for culturally diverse groups must be addressed, with the first and most obvious step to increase recruitment of diverse participant groups in neurobiological and treatment studies.

A number of biological, cultural and developmental factors have been identified previously that are either implicated or known risk factors in the aetiology of EDs [9]. However, it is important to note that the single biggest risk factor for developing an ED is being female [11]. Culbert and colleagues [12] published an extensive integrative review of these risk factors, proposing a transactional process that unfolds between biological vulnerabilities, environmental factors and sociocultural risk factors, leading to the onset of an ED.

A number of treatments have been identified to be moderately effective for BED, such as cognitive–behavioural, interpersonal psychotherapy, structured self-help treatment and psychotropic medications [13]. In a recent umbrella review of meta-analyses of treatment outcomes transdiagnostically, it was found that the strongest evidence existed for lisdexamphetamine (LDX) and antidepressants, followed by general psychotherapy and behavioural treatments, with cognitive therapy for ED symptoms performing least effectively [13]. Clear evidence also exists that highlights the divergent neurobiological characteristics of individuals with BED compared to those with obesity, for example, in the differential response to pharmacological treatment targeting impulsivity/compulsivity and reward processing [14]. Irrespective of the type of therapy, it is clear that pharmacological treatments provide targeted treatment for individuals with BED [14].

In the context of the significant burden of illness [15] for individuals and their families with BED, treatment effectiveness especially in a medium-to-long-term timeframe, is a critical consideration. In a recent meta-analysis on the long-term effectiveness of psychological and medical treatments for BED, effectiveness of treatment, defined as reduced/abstinence from binge episodes and reduced EDs and general psychopathology, data supported the medium-term effectiveness of psychotherapy, structured self-help treatment and combination treatment, e.g., pharmacotherapy and psychotherapy [15]. However long-term effectiveness beyond 12 months post treatment is infrequently reported, and data are inconsistent [15]. Thus, new ideas for testable treatments are crucial to improve outcomes and lower the personal, family, occupational, economic and community burden of BED.

## 2. Neuroimaging and BED

BED has been conceptualized as an impulsive/compulsive ED with altered reward sensitivity and food-related attentional biases [16,17]. Specifically, individuals with BED move from a ventral striatal reward-based mode of reward-related food consumption to a dorsal striatal impulsive/compulsive mode of reward-related food consumption [16,17]. A systematic review by Leenaerts and colleagues [18] examining the neurobiological reward system and binge eating demonstrated that in a resting state, individuals who binge eat exhibit a lower striatal dopamine release, a change in the volume of the striatum, frontal cortex and insula, as well as a lower frontostriatal connectivity. Conversely, when performing a task, people who binge eat demonstrate a higher activity of the brain reward system when anticipating or receiving food, more model-free reinforcement learning and more habitual behavior [18]. There is also evidence to support the incentive sensitisation theory of binge eating, which suggests that repeated BE episodes sensitise the brain, which leads to a higher incentive salience, or ‘wanting’ of food [18]. This sensitisation process occurring neurologically could explain why greater activity is seen in regions associated with reward when anticipating food but not in anticipation of other potentially rewarding items, such as money [18]. Evidence is also emerging that individuals who binge eat demonstrate hyperactivity of the anterior cingulate cortex and insula when anticipating food rewards [18].

Current evidence suggests that one neurobiologically based etiological factor observed in BED may be a maladaptation of the corticostriatal circuitry, which is responsible for regulating motivation and impulse control in response to salient cues, similar to that found in other impulsive/compulsive disorders [16]. More specifically, diminished activity in regions associated with self-regulation, the frontostriatal circuits, may drive the recurrent OBEs seen in BED [17]. There is believed to be a structural and functional disconnect between the frontal cortex and the striatum, which may lead to an increased likelihood of habitual, repetitive behaviours [18]. These differences are seen as a neurological parallel individuals with obsessive–compulsive disorder. However, it must be highlighted that the neuroimaging findings reported in individuals who binge eat are the result of a range of neurobiological interactions; it is important to take a holistic perspective of the known aberrances rather than emphasising the singular importance of one region.

A pattern of diminished neural responsivity was found in a recent fMRI study published by Donnelly and colleagues [19], specifically in individuals with BED viewing high-energy, highly palatable foods. Diminished neural responsivity to foods consistent with those consumed during a binge episode was found not only in the frontostriatal circuits but also across all neural regions examined. One possible explanation is that there is a mechanism of reduced satiation demonstrated by the diminished neural responses to food stimuli in fMRI, together with an imbalance of the frontostriatal reward system and control-related circuit abnormalities in those with BED, which informs the intensity and frequency of binge episodes [19].

## 3. Genetic Variables

In the last twenty years, a growing body of evidence has been published offering considerable advancement in elucidating the role of genetic factors in the aetiology of EDs. Earlier research, such as a large-scale twin studies of EDs, has been conducted over the past two decades, which contributes significantly to this area; twin studies have demonstrated significant heritability of binge eating [20,21]. Twin studies have demonstrated that the heritability reported for the specific symptom of binge eating with no compensatory behaviours is between 41 and 57%, with the remaining variance attributable to environmental factors [22].

Although ED symptoms are reported to be moderately heritable [23], to date, most-genome-wide association studies (GWASs) investigated anorexia nervosa (AN). In a recent study examining the polygenic relationship between psychiatric disorders and anthropometric traits, the results demonstrated that several ED symptoms, including binge eating and body dissatisfaction, were significantly associated with psychiatric and anthropometric polygenic scores (PGSs) [24]. In particular, individuals with binge-type eating disorders had higher polygenic scores than the controls for other psychiatric disorders, including depression, schizophrenia, and attention-deficit hyperactivity disorder, and higher polygenic scores for body mass index [1]. Existing research suggests that sub-threshold ED symptoms may be partially etiologically related (i.e., psychiatric and anthropometric origins), but that metabolic genetic factors may differentiate between symptoms and threshold EDs [24,25].

### Gene Variants

Ideally, the treatment of eating disorders occurs as soon as possible after symptoms have been identified. However, with the advances in molecular testing, it is important to consider that broad, early testing in pre-adolescence could identify individuals who have higher susceptibility to the onset of an ED, which could inform targeted prevention to reduce the risk of onset [26]. Eating disorders are understood to develop as a result of the intersection of genetic factors and environment; so, it is critical that ongoing research continues in an effort to broaden the understanding of these illnesses, in addition to reducing stigma regarding the reasons for disorder onset.

The current understanding from twin studies demonstrates that the genetic heritability of BED ranges from 41 to 57% [22]. There is a range of specific genetic polymorphisms documented in BED, including three serotonergic genes, *5-HTT*, *5-HT2C* and *5-HT2A* [26]. The 5-HTT gene has been of considerable interest; it is a serotonin transporter that is known to influence personality variables such as happiness, subjective wellbeing, and mental health disorders including BED [27]. Dopaminergic genes, including *DRD2*, *ANKK1*, *OPRM1*, *COMT*, *DAT1* and *DRD3*, especially a polymorphism called *Taq1A* of *DRD2*, have been associated with higher reward sensitivity and obesity [28,29,30], which is of significant interest in the effective treatment of BED.

A further goal for those seeking the diagnosis and treatment of an eating disorder is obtaining individual genetic information, which could contribute to improved clinical management and individualised treatment [26]. One hypothesis is that neurobiological processes underlying BED may include a hyperreactivity of the immune system, which leads to a dysfunction in neuropeptide production [26]. Therefore, pharmacogenetic tests that detect polymorphisms in coding for enzymatic metabolism of psychotropics in common use (e.g., antidepressants) are in current clinical use to aid in the identification of individual risk for adverse events from current (e.g., antidepressants) and future medications (e.g., the *GLP1* agonists) that may reduce OBEs in people with recurrent binge eating [25].

## 4. Pharmacological Treatments

Pharmacological interventions, specifically neurostimulant LDX, have firmly emerged as a potential additional form of treatment to augment cognitive therapy for the treatment of BED. It is important that research continues to explore how LDX can be utilised in the treatment of BED, due to evidence that clearly highlights how this medication rapidly reduces binge episode frequency, impulsive eating behaviour and obsessive thinking related to food in individuals with BED [31]. Formal randomised control trial studies are required to establish the precise contribution LDX could make to the improved treatment outcomes of people with BED.

More research is also needed into the mechanism of action of medications with known efficacy in BED. LDX is currently the only medication approved for the treatment of moderate to severe BED; however, explanations regarding its specific neurological mechanism are complex. For example, a recent study by Griffith and colleagues [31] investigated the neural mechanism of symptom improvement via LDX in individuals with moderate to severe BED who underwent baseline and 8-week follow-up functional magnetic resonance imaging (fMRI). The functional connectivity in a range of networks believed to underlie BED was examined pre–post LDX treatment. Over 97% of the participants experienced a remission or reduction in BED symptoms after LDX pharmacological treatment; however, the fMRI data revealed an unexpected pattern of change, i.e., that there was a limited overlap between the connectivity of nodes associated with improvement following LDX and those in which the BED group differed from the control group [31]. This research highlighted that the LDX pharmacological treatment appears not to act via neural networks associated with pre-treatment diagnostic features; rather, connectivity with the interoceptive network, which allows individuals to identify an awareness of their internal physiological and emotional state, is implicated in the core symptom of a loss of control seen in BED and is a target of treatment [31].

Evidence is emerging on the use of the noncompetitive N-methyl-D-aspartate receptor (*NMDAr*) antagonist ketamine as a novel psychopharmacotherapy for transdiagnostic EDs, including BED [32,33]. The increasing interest in using ketamine in the treatment of EDs is related to its known function of reducing a range of behavioural and affective symptoms among individuals with treatment-resistant psychiatric illness, together with the evident need to identify effective pharmacological treatment alternatives for EDs [32].

Psychedelic-assisted therapy (PAT) was previously investigated in relation to patient response and treatment acceptability for the treatment of mood disorders, addiction and post-traumatic stress disorder (PTSD) [34]. To our knowledge, only one study [35] examined the use of psychedelics, specifically, 3,4-methylenedioxymethamphetamine (MDMA)-assisted therapy (MDMA-AT), for the treatment of EDs with comorbid PTSD. The results highlighted that MDMA-AT significantly reduced eating disorder symptoms in individuals with BED and comorbid PTSD, which is thought to be related to the anxiolytic and prosocial effects, as well as the facilitation of socio-emotional processing, of MDMA [35].

## 5. The Gut–Brain Axis (GBA)

The gut–brain axis (GBA) is a bidirectional mechanism of communication that connects the central and the enteric nervous systems, linking emotional and cognitive areas of the brain with peripheral intestinal function in the gut [36]. It is a key area of cross-discipline research between physical and mental health, with a significant amount of research interest in recent years due to its apparent role in a number of medical and psychiatric illnesses [37,38]. A key outcome in this area of research is the quantifiable diversity in terms of number, abundance and distribution of microbes within the gut [37], which are known to be altered in individuals who engage in overeating [38].

A recent animal model study of the microbiota gut–brain axis in mice demonstrated that alterations in the intestinal microbiota were responsible for the excessive intake of palatable foods in mice [38]. Stress, together with a history of dieting, causes significant changes in the microbiota and the intestinal metabolism, which disinhibit the vagus nerve terminals in the gut [38]. In turn, this may lead to a subsequent hyperactivation of the gut–brain axis passing through the vagus, the solitary tract nucleus and the paraventricular nucleus of the thalamus. In this study, faeces from healthy mice including the microbe *Faecalibacterium prausnitzii* were transplanted into overeating mice, which was reported to improve the activity of the gut–brain pathway, alleviating excessive food intake [38].

### Gut Microbiota and BED

The human gut microbiome is thought of as our second brain and has emerged as a potential key factor in the aetiology of mental and physical disorders [37]. A well-established relationship exists between the gut microbiota and general mental health. For example, in a randomised, double-blind controlled trial, the probiotic supplementation (*L. reuteri PBS072* and *B. breve BB077*) of new mothers significantly reduced the risk of the onset of low mood, anxiety, stress and postpartum depression 45 and 90 days postpartum [39]. Clinical research on the gut microbiome in the context of EDs, including BED, is an emerging area of significant clinical interest. Terry and colleagues [40] completed a recent critical analysis of the role of the gut microbiome in EDs. The authors concluded that, by increasing the population of the gut microorganisms *Lactobacilli* spp., *Bifidobacterium* spp. and *Enterococcus* spp., ED symptoms are likely to improve, with associated improved clinical outcomes. In general, by increasing the diversity of our gut microbiome, it is hoped to improve symptoms of anxiety and depression, as well as weight regulation for those who need to consider this factor as part of their treatment [40].

The diversity of microbes in an individual’s microbiome is known as α-diversity and is commonly examined in studies in this area; increased α-diversity has been found to be positively correlated with better health [37]. A decrease in the diversity of the gut microbiota is reported to be linked with increased anxiety, depression and ED psychopathology [38,40]. An imbalance in the gut microbiome, leading to inflammation or ‘gut microbiota dysbiosis’, can result from dieting or a reduction in macronutrient availability and is associated with physical and mental ill health, often resulting from an overgrowth of potentially harmful microorganisms, the loss of beneficial microorganisms, and a reduction in species diversity resulting in the loss of the normally tolerogenic and symbiotic relationship [40]. Earlier work highlighted that microbiota homeostasis is an essential factor for healthy communication within the gut–brain network [41], alongside optimal energy regulation [42] and fat storage [43]. Additionally, gut microbiota dysbiosis was correlated with intestinal inflammation, gut permeability and may trigger immune reactions in the regulation of hunger/satiety, which, in turn, may amplify certain ED symptoms such as binge eating [44].

## 6. Integrating Neurobiological Findings: Future Treatments

BED is an underrecognized and undertreated condition. Potentially due to a partial understanding of the aetiology of BED, existing evidence-based treatments remain inadequate, with individuals likely to experience numerous relapses in the trajectory of their ED [40]. Moreover, people seeking treatment for BED are more likely to receive treatment for weight loss than evidence-based psychological treatment, which contributes to long-term health implications and health system burden [10].

Neuropsychological and genomic assessments need to be developed to enable the tailoring of individual treatments for people with BED. This would optimise the treatment outcomes through identifying individual variances in aspects of cognition related to the treatment of an eating disorder. For example, by completing a systematic neuropsychological assessment including executive function, cognitive flexibility, problem solving and holistic thinking, individuals could be provided with specific information regarding their individual strengths and weaknesses and how treatment is tailored to provide client-centred care. Furthermore, the known differences on a neurological level in relation to BED must be integrated into treatment, to empower, educate and reduce stigma. For example, the structural and functional differences documented in reward processing, such as heightened sensitivity to highly palatable foods, greater model-free learning and heightened likelihood of engaging in repetitive, habitual behaviours when eating, could be used to educate individuals about this mental illness at a bare minimum, which would reduce stigma and shame about the aetiology of this illness. Further to this, the formal neurobiological assessment of these known differences could serve to inform an individualised treatment approach, particularly when considering an individual’s case formulation and environmental vulnerabilities to binge episodes [18,19,31].

Understanding the genetic component in the aetiology of eating disorders is critical. Considering current knowledge highlights that EDs develop as a result of many factors, including genetic heritability, Michael and colleagues [45] propose that individuals with EDs fall within the scope of practice for genetic counselling. However people with EDs are rarely referred for genetic counselling, despite clear indications for receiving this form of treatment [45]. In the treatment of any individual with an ED, genetic counselling could improve the treatment outcomes by providing clear, scientific, evidence-based accurate information regarding the risk of recurrence, answer questions regarding heritability to inform family-planning decisions and reduce feelings of internalised shame, distress, guilt and stigma [45]. Individuals with BED referred for treatment could receive genetic counselling prior to commencing cognitive or interpersonal treatment, which could increase the benefit of support-seeking and treatment and encourage recovery [45].

There is emerging evidence that the microbiota–gut–brain axis may mediate energy balance, highlighting a possible underlying pathogenesis factor of BED and providing potential therapeutic strategies [38]. It is clear that the gut microbiome is an area of great interest with promising findings that have the potential to be applied clinically to improve ED recovery [40]. A restored microbial balance presents a possible treatment target for EDs including BED, where gut microbiota dysbiosis and appetitive changes are especially relevant [44]. Probiotic supplementation presents an exciting area of research that could be explored further in the area of eating disorders, with the aim of improving the gut microbiota and eubiosis.

To improve the accessibility for individuals with BED to early, low- or no-cost, evidence-based treatment, internet-based guided self-help (GSH) presents a promising alternative to standard interpersonal psychotherapy. Wyssen and colleagues [46] recently reported on a randomised clinical controlled trial of an eight-session GSH treatment program for BED based on cognitive–behavioural therapy. The results highlighted high treatment satisfaction and high efficacy, with a significant reduction in binge eating episodes from a mean of 3.4 to a mean of 1.7 per week [46]. Carrard and colleagues [47] reported similar findings in an earlier study examining the acceptance and efficacy of a six-month GSH treatment program for obese BED patients. The results highlighted significantly reduced binge eating episodes and improved quality of life for participants who completed the treatment.

## 7. Conclusions

BED is common and burdensome; however, present and future advances in neurobiology present opportunities to develop tailored treatments and improve prevention. Further research is needed to close the gaps in our understanding of combined treatment interventions. In this paper, we conceive a future where assessments will incorporate a neurocognitive and genomic profile, thus going beyond symptoms and diagnostic features. This would apply as well to those who present in early life years with a neuropsychiatric/endocrine risk and associated factors for BED such as deficits in attention, impulsivity and hunger/satiety dysregulation. Mental health care has been slow in translating the neuroscientific understanding of determinants of BED and it is time to do so. The integration of genetic testing and tailored counselling as a precursor to cognitive therapy may offer an alternative modality of treatment, which could encourage ongoing treatment engagement and recovery for those affected by BED. Evidence remains small regarding the use of emerging pharmacological treatments, such as ketamine and psilocybin; however, further studies are indicated, as these treatments are likely to be most effective for individuals with treatment-resistant forms of BED.

## Data Availability

Not applicable.
